# Multistate outbreaks of *Salmonella* infections linked to imported Maradol papayas – United States, December 2016–September 2017

**DOI:** 10.1017/S0950268819001547

**Published:** 2019-09-10

**Authors:** R. Hassan, B. Whitney, D. L. Williams, K. Holloman, D. Grady, D. Thomas, E. Omoregie, K. Lamba, M. Leeper, L. Gieraltowski, the Outbreak Investigation Team

**Affiliations:** 1 Centers for Disease Control and Prevention (CDC), Atlanta, Georgia, United States; 2 CAITTA, Inc., Herndon, Virginia, United States; 3 United States Food and Drug Administration, College Park, Maryland, United States; 4 Maryland Department of Health, Baltimore, Maryland, United States; 5 Virginia Department of Health, Richmond, Virginia, United States; 6 New York State Department of Agriculture and Markets, Albany, New York, United States; 7 New Jersey Department of Health, Trenton, New Jersey, United States; 8 New York City Department of Health and Mental Hygiene, New York City, New York, United States; 9 California Department of Public Health, Richmond, California, United States

**Keywords:** Food-borne infections, outbreaks, salmonellosis

## Abstract

Foodborne salmonellosis causes approximately 1 million illnesses annually in the United States. In the summer of 2017, we investigated four multistate outbreaks of *Salmonella* infections associated with Maradol papayas imported from four Mexican farms. PulseNet initially identified a cluster of *Salmonella* Kiambu infections in June 2017, and early interviews identified papayas as an exposure of interest. Investigators from Maryland, Virginia and Food and Drug Administration (FDA) collected papayas for testing. Several strains of *Salmonella* were isolated from papayas sourced from Mexican Farm A, including *Salmonella* Agona, Gaminara, Kiambu, Thompson and Senftenberg. Traceback from two points of service associated with illness sub-clusters in two states identified Farm A as a common source of papayas, and three voluntary recalls of Farm A papayas were issued. FDA sampling isolated four additional *Salmonella* strains from papayas sourced from Mexican Farms B, C and D. In total, four outbreaks were identified, resulting in 244 cases with illness onset dates from 20 December 2016 to 20 September 2017. The sampling of papayas and the collaborative work of investigative partners were instrumental in identifying the source of these outbreaks and preventing additional illnesses. Evaluating epidemiological, laboratory and traceback evidence together during investigations is critical to solving and stopping outbreaks.

## Introduction

Nontyphoidal salmonellosis is the most common bacterial cause of foodborne illness in the United States, leading to approximately 1 million domestically acquired foodborne illnesses, 19 000 hospitalisations and 380 deaths annually [1]. Most people infected with *Salmonella* develop diarrhoea, fever and abdominal cramps that usually lasts 4–7 days [2]. Young children, older adults and immunocompromised people are most likely to have severe infections [2].

Public health laboratories in the United States conduct serotyping and subtyping on *Salmonella* isolates by pulsed-field gel electrophoresis (PFGE). These PFGE patterns are uploaded to PulseNet, the national molecular subtyping network for foodborne disease surveillance, and are monitored to detect clusters of illness with indistinguishable PFGE patterns. Isolates with indistinguishable PFGE patterns are more likely to share a common source [3, 4].

The proportion of foodborne outbreaks, including *Salmonella* outbreaks, attributed to raw produce, such as fruits and vegetables, has been increasing in the United States since the 1970s [5–8]. Approximately half of the fresh fruit and about 20% of the fresh vegetables consumed in the United States are imported from other countries, most of which come from Mexico [9, 10]. There have been several multistate *Salmonella* outbreaks attributed to imported raw fresh produce over the past decade [11–18]. The implicated foods include cucumbers, mangoes, papayas, hot peppers and cantaloupe. Most of these foods were imported from Mexico, although two outbreaks were associated with produce imported from other countries in Central America [11–18].

After an outbreak of *Salmonella* infections in 2011 was linked to imported papayas from Mexico and a large proportion of import sampling identified *Salmonella*, the US Food and Drug Administration (FDA) issued a broad import alert to detain all whole, fresh papayas entering the United States from Mexico [12, 19]. To be removed from import alert status and supply Mexican papayas to the United States, firms need to provide documentation with sufficient evidence that future shipments of their papaya will not be adulterated; FDA may consider five consecutive commercial shipments over a period of time, analysed for *Salmonella* as prescribed by the FDA, as being adequate to be added to the ‘green list’ [19]. The 2011 import alert was still in effect when the outbreaks described below occurred; however, suppliers of Mexican papayas available in the United States at the time had been through the ‘green list’ process.

In this paper, we report the results of four multistate outbreaks of *Salmonella* infections linked to imported Maradol papayas from Mexico.

## Methods

### Outbreak detection

In June 2017, PulseNet detected a cluster of 13 *Salmonella* Kiambu infections with an indistinguishable PFGE *Xba*I restriction enzyme pattern. This pattern is the most common *Salmonella* Kiambu pattern in the PulseNet database. However, *Salmonella* Kiambu is a rare serotype, and the number of isolates uploaded to PulseNet in June 2017 was higher than expected based on data from previous years. State and local health departments and the Centers for Disease Control and Prevention (CDC) initiated a multistate investigation to determine the source of the outbreak and prevent additional illnesses.

### Initial case definition and case finding

Additional illnesses were identified from isolate uploads to PulseNet. The initial outbreak case definition was an infection with the outbreak strain of *Salmonella* Kiambu in a person with illness onset on or after 10 April 2017.

### Epidemiological investigation

Local and state investigators interviewed case-patients with routine enteric disease questionnaires to gather exposure information. Due to the increasing number of uploads to PulseNet and to gather more detailed exposure information, CDC requested that local and state health departments re-interview case-patients with the national hypothesis generating questionnaire (NHGQ), which includes over 300 questions on various food, animal and other exposures in the week prior to illness onset. Based on initial exposure information received, CDC developed a modified NHGQ to include detailed questions on Hispanic-style foods and eventually a focused questionnaire to collect details for foods of most interest in the outbreak. Food exposures were compared with expected background rates for foods reported by healthy people interviewed in the 2006–2007 FoodNet Population Survey, using the binomial test [20].

### Traceback investigation

State and local regulatory agencies collected invoices and records from points of service where case-patients reported eating or purchasing food items of interest, and the FDA conducted traceback investigations to trace these items to their original source. Traceback investigations were focused on illness sub-clusters: locations where two or more unrelated case-patients reported eating or purchasing suspected food items. Traceback investigations involve a thorough review of product distribution records and supporting information to identify or rule out a common supplier or source for the food item of interest across sub-clusters.

### Laboratory investigation

State public health laboratories serotyped and subtyped clinical *Salmonella* isolates by PFGE using standard methodology [18, 19]. State and local public health laboratories and CDC's enteric disease laboratory further characterised a sub-set of clinical isolates by whole-genome sequencing (WGS). Genomic DNA was extracted using a QIAGEN DNeasy Blood and Tissue Kit (Qiagen, Aarhus, Denmark). The DNA libraries were prepared using a Nextera XT DNA Library Preparation Kit (Illumina, San Diego, CA) and the DNA sequencing was performed on the Illumina MiSeq Sequencing System using the 2 × 250 base pair sequencing chemistry. High-quality single nucleotide polymorphism (hqSNP) analysis was performed using the Lyve-SET pipeline [21]. Depending on the serotype, either closed PacBio sequences or draft Illumina assemblies were used as references with prophages removed from the analysis. Read mapping was performed using SMALT and SNPs were called using VarScan at >20× coverage, >95% read support and clustered SNPs <5 base pair apart were filtered out.

Local and state investigators collected samples of suspected food items from grocery stores and distribution centres for *Salmonella* testing. FDA also collected samples of suspected foods at domestic distributors and at import as products were entering the country. State and local public health and regulatory laboratories and FDA laboratories tested these food products for *Salmonella* and further characterised any *Salmonella* isolates identified by serotype, PFGE and WGS.

## Results

### Epidemiological investigation

By 20 July, 18 of 30 (60%) case-patients identified themselves as Hispanic or Latino, and common foods reported from the NHGQ data received from 24 case-patients included mangoes (50%), papayas (42%), cilantro (35%), fresh hot peppers (26%) and sprouts (22%). These frequencies were compared with consumption rates reported by healthy Hispanic people in the 2006–2007 FoodNet Population Survey in the months of May and June in the week before they were interviewed or to consumption rates reported by Hispanic ill people in CDC's NHGQ database, which includes NHGQs collected for cluster cases or sporadic illnesses [20]. The reported frequencies among case-patients for mangoes (50% *vs.* 34%), papayas (42% *vs.* 16%) and sprouts (22% *vs.* 7%) were statistically significantly higher than the expected background rates, while the frequencies for cilantro (35% *vs.* 48%) and fresh hot peppers (26% *vs.* 17%) were not significantly higher than expected.

On 5 July, New York City notified CDC of an illness sub-cluster including two ill people who were residents of the same nursing home prior to their illness onsets. One of these ill people was a confirmed case-patient in the outbreak, and the other had reported *Salmonella* infection with sub-typing pending. The nursing home served papayas and mangoes regularly to the residents. However, we were unable to confirm definitively when or if papayas or mangoes were consumed by either case-patient.

On 13 July, Maryland investigators reported to CDC a second potential illness sub-cluster where five ill people reported shopping at the same international grocery store prior to their illness. Three of these ill people were confirmed case-patients in the outbreak, and two others had reported *Salmonella* infections with sub-typing not yet completed. These ill people had illness onset dates ranging from 31 May to 26 June. Among three ill people with purchase information, the only common food purchased from this store was papayas.

### Traceback investigation

**Fig. 1.**
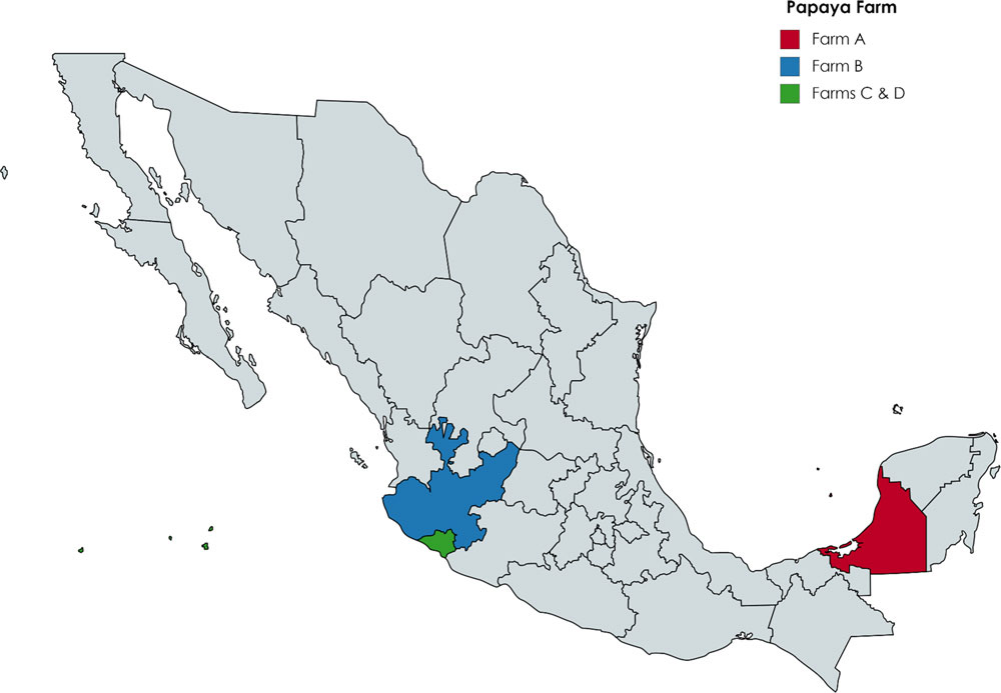
Farm locations of papaya samples yielding *Salmonella* outbreak strains, by state, Mexico, 2016–2017.

State regulatory agencies and FDA conducted traceback for the papayas purchased and served at the two sub-cluster locations in New York City and Maryland. Investigators found that both locations sold or served fresh Maradol papayas from Mexico, and both received papayas from the same Mexican farm: Farm A (Fig. 1). The investigation did not identify a single shipment of interest. Preliminary traceback documents related to mangoes reportedly purchased by case-patients were also collected; however, traceback for mangoes was not completed once papayas were confirmed as the vehicle based on multiple product samples and traceback convergence.

### Laboratory investigations

On 12 July, Maryland investigators collected 10 single, whole papayas for *Salmonella* testing from the sub-cluster store location in Maryland: five yellow Maradol papayas from Mexico and five green papayas from Guatemala. The two papaya varieties were stored in separate bins at the store. *Salmonella* was isolated from three of the five Mexican Maradol papayas that were sampled, which originated from Farm A in Mexico; no *Salmonella* was identified in the five green papayas from Guatemala. Four separate *Salmonella* serotypes were identified: Kiambu, Thompson, Gaminara and Senftenberg. The *Salmonella* Kiambu strain identified differed from the clinical isolates in the outbreak by PFGE by 2–3 bands; this strain was very rare and had not been seen in the PulseNet database since 2012. The *Salmonella* Thompson and *Salmonella* Gaminara strains were both new to the PulseNet database, and the *Salmonella* Senftenberg strain was very rare.

On 20 and 24 of July, investigators in Virginia collected seven single, whole Maradol papayas from two Virginia grocery store locations that received papayas from the same distributor as the Maryland sub-cluster grocery store location. *Salmonella* was isolated from five of these papayas, which were all collected from the same store location and originated from Farm A in Mexico. Three strains were identified: the same strain of *Salmonella* Gaminara that had been identified previously, as well as a strain of *Salmonella* Agona, and a second variant strain of *Salmonella* Thompson.

From July through August 2017, FDA collected and tested 13 targeted samples of Maradol papayas as part of the outbreak investigation, including both domestic and import samples. The sampling targeted papaya firms based on preliminary traceback and state laboratory information. In total, there were 185 single, whole Maradol papayas that made up the 13 targeted samples, and each sample consisted of between 10 and 30 single papaya subsamples. *Salmonella* was isolated from seven of these samples. Five *Salmonella* strains were identified in four FDA samples of papayas originating from Farm A in Mexico, including the original *Salmonella* Kiambu outbreak strain found in clinical isolates, as well as the same *Salmonella* Thompson, *Salmonella* Agona and *Salmonella* Senftenberg strains isolated from papayas collected in Maryland and Virginia. FDA testing also isolated two additional strains of *Salmonella* Senftenberg from Maradol papayas. One strain was very rare, last seen in the PulseNet database in 2011, and the other strain was new to the PulseNet database. All papaya sampling was conducted on the surface of the fruits. Since pulp analysis was not conducted, it is unknown if the contamination migrated to the interior of the fruit.

Investigators in New Jersey and New York collected nine single Maradol papaya samples for testing; none of these yielded *Salmonella.* In total, *Salmonella* was isolated from 8 of 26 (31%) single papayas collected by state and local investigators and in seven of 13 targeted papaya samples collected by federal investigators.

From late July to early August, state investigators collected 25 samples of mangoes from retail locations where case-patients reported purchasing them in Maryland (12), New Jersey (7), New York (3) and Virginia (3). No mangoes samples tested yielded *Salmonella*.

### Summary of final outbreak findings

As additional *Salmonella* strains were isolated from papaya samples, the PulseNet database was queried for recent clinical isolates that were indistinguishable from the identified patterns. Based on laboratory and epidemiological evidence, strains identified in papaya samples originating from Farm A in Mexico were added to the case definition. The final case definition was an infection with one of the identified outbreak strains in a person with illness onset on or after 1 May 2017. In total, we identified 213 outbreak-associated cases of *Salmonella* Kiambu (50 cases), *Salmonella* Thompson (142 cases), *Salmonella* Agona (12 cases), *Salmonella* Gaminara (7 cases) and *Salmonella* Senftenberg (2 cases) (Table 1). Case-patients were from 22 states: Connecticut (6), Delaware (4), Florida (1), Georgia (1), Iowa (2), Illinois (6), Kentucky (4), Massachusetts (10), Maryland (11), Michigan (1), Minnesota (4), North Carolina (6), New Jersey (41), New York (70), Ohio (2), Oklahoma (5), Pennsylvania (8), South Carolina (1), Tennessee (2), Texas (9), Virginia (18) and Wisconsin (1).

**Table 1.** Number of outbreak-associated clinical and papaya isolates by Mexican farm and *Salmonella* strain, 2016–2017

	Onset date	Serotype	PFGE pattern	Number of clinical isolates	Number of papaya isolates
Farm A	17 May–20 September 2017	Agona	JABX01.0258	12	17
		Gaminara	TDJX01.0463	7	16
		Kiambu	TENX01.0003	50	4
			TENX01.0049	0	3
		Senftenberg	JMPX01.0157	5	15
			JMPX01.0296	0	1
			JMPX01.0526	0	1
		Thompson	JP6X01.0757	55	1
			JP6X01.0838	87	6
Farm B	23 July–14 August 2017	Urbana	JQGX01.0194	7	6
Farm C	19 July–7 August 2017	Infantis	JFXX01.1235	1	1
		Newport	JJPX01.5863	3	2
Farm D	20 December, 2016–16 August 2017	Anatum	JAGX01.0013	20	4
			JAGX01.0721	0	1
Unrelated farm^a^	N/A	Abaetetuba	JREX01.0011	0	1

a This farm was not associated with any identified illnesses or outbreaks during the investigations described in this paper.

**Fig. 2.**
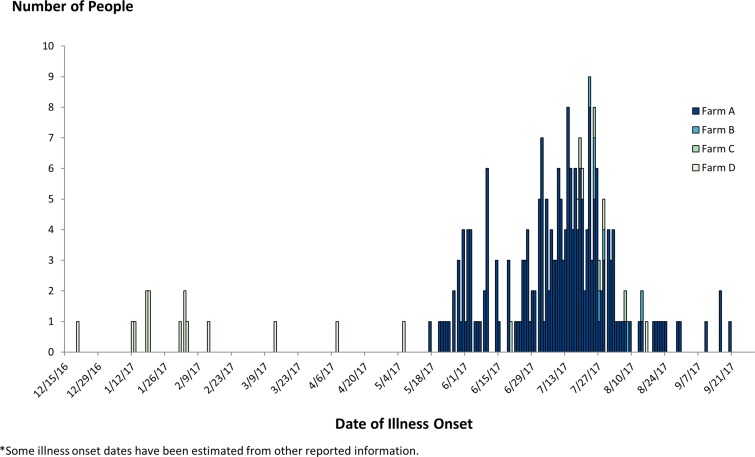
People infected with the outbreak strains of *Salmonella* (*n* = 244), by date of illness onset and papaya farm, United States, 2016–2017. *Some illness onset dates have been estimated from other reported information.

Case-patients ranged in age from <1 to 95 years (median: 40), and 62% were female. Among 169 case-patients with information, 68 (40%) were hospitalised and one died. Illness onset dates ranged from 17 May to 20 September 2017 (Fig. 2). In total, among 168 case-patients with race/ethnicity information, 113 (67%) identified as Hispanic or Latino. Exposure information was available for 143 case-patients, of whom 78 (55%) reported consuming fresh papaya in the week prior to illness. This proportion was significantly higher than results from the 2006–2007 FoodNet Population Survey, where 22% of healthy Hispanic people reported eating papayas in the summer months in the week before they were interviewed [20]. No other food items were reported by case-patients significantly more than expected compared with background consumption rates.

### Identification of additional outbreaks

Among the seven targeted papaya samples collected by FDA which yielded *Salmonella*, three did not originate from Farm A in Mexico. Four *Salmonella* strains were identified in these papaya samples, all of which were new or very rare in the PulseNet database.

One rare strain of *Salmonella* Abaetetuba was identified, which had only been seen in PulseNet once previously from a papaya sample originating from Mexico in 2011. No clinical isolates were identified with this strain. The other three *Salmonella* strains identified from FDA's targeted testing led to the identification of additional papaya-associated outbreaks of salmonellosis, after recent clinical isolates with these strains were found in PulseNet. Additionally, FDA non-targeted, increased surveillance sampling identified two strains of *Salmonella* Anatum that were ultimately linked to a previously unsolved outbreak. In total, testing identified 15 strains of *Salmonella* from papaya samples associated with five different Mexican farms (Table 1).

### Outbreak of *Salmonella* Urbana infections

FDA targeted sampling identified a strain of *Salmonella* Urbana in papayas from Farm B in Mexico (Fig. 1). This pattern was new to the PulseNet database and had not been seen prior to the summer of 2017. In total, we identified seven *Salmonella* Urbana outbreak-associated cases from three states: New Jersey (5), New York (1) and Pennsylvania (1). Case-patients ranged in age from <1 to 57 years (median: 1), and 4 (57%) were female. Illness onset dates ranged from 23 July to 14 August 2017. Five (71%) of the seven case-patients were of Hispanic ethnicity. Four (57%) reported being hospitalised and no deaths were reported. Among five case-patients with exposure information, three (60%) reported consuming fresh papaya in the week prior to their illness.

### Outbreak of *Salmonella* Newport and *Salmonella* Infantis infections

FDA targeted sampling identified strains of *Salmonella* Newport and Infantis in papayas from Farm C in Mexico (Fig. 1). The strain of *Salmonella* Newport was very rare and was last seen in PulseNet in 2006, and the *Salmonella* Infantis strain was new to the database and had not been seen prior to the summer of 2017. In total, we identified four outbreak-associated cases of *Salmonella* Newport (3 cases) and *Salmonella* Infantis (1 case) from 4 states: Illinois (1), Massachusetts (1), Michigan (1) and New York (1). Case-patients ranged in age from 40 to 82 years (median: 63), and 2 (50%) were female. Two (50%) of the four case-patients were hospitalised and no deaths were reported. Illness onset dates ranged from 19 July to 7 August 2017. Two (50%) of the case-patients were of Hispanic ethnicity. Among three case-patients with exposure information, all three (100%) reported consuming fresh papaya in the week prior to their illness.

### Outbreak of *Salmonella* Anatum infections

In August, *Salmonella* Anatum was identified by FDA in a non-targeted, increased surveillance sample from an imported papaya from Farm D in Mexico (Fig. 1); the collection of this sample was not directly related to the previously described outbreak investigation. This strain of *Salmonella* was indistinguishable from an unsolved *Salmonella* Anatum outbreak from the spring of 2017, in which papayas had been the suspected source of the outbreak at the time based on epidemiological evidence. While FDA determined that 16 different papaya suppliers in Mexico could have accounted for product purchased by several case-patients in this outbreak, the traceback investigation was limited due to the lack of sub-clusters. As the traceback findings could not identify a specific supplier due to limited papaya exposure information and limited lot code documentation throughout the distribution chain, no conclusions or actionable findings could be drawn; therefore, the investigation for this *Salmonella* Anatum outbreak ended in May of 2017.

After the product testing results identified the outbreak strain, the investigation was re-opened and additional related illnesses were identified. In total, we identified 20 cases of *Salmonella* Anatum from three states: Arizona (1), California (18) and Colorado (1). Case-patients ranged in age from <1 to 89 years (median: 43), and 85% were female. Among 11 case-patients with available information, five (45%) were hospitalised. One death was reported from California. Illness onset dates ranged from 20 December 2016 to 16 August 2017. Among 11 case-patients with information, 10 (91%) were of Hispanic ethnicity. Ten (91%) of 11 case-patients interviewed reported eating papayas in the week prior to their illness.

**Fig. 3.**
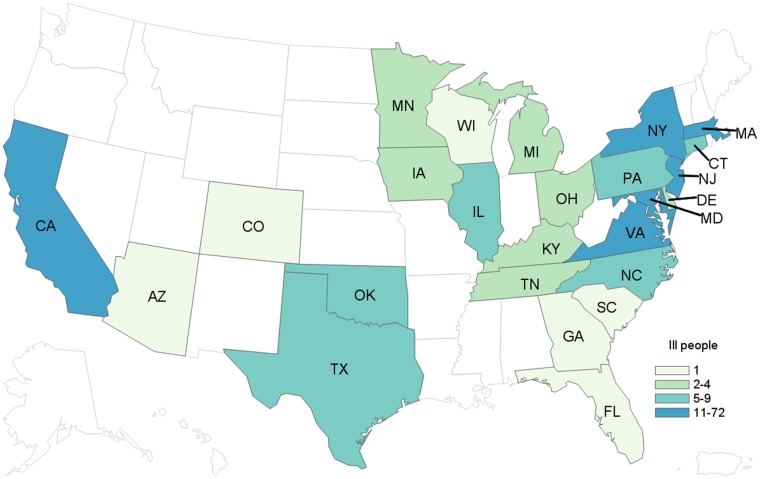
People infected with the outbreak strains of *Salmonella* (*n* = 244), by state of residence, United States, 2016–2017.

In total, we identified 244 cases from 25 states across all four outbreaks, with illness onset dates ranging from 20 December 2016 to 20 September 2017 (Fig. 3).

### Whole-genome sequencing

WGS was completed on 191 clinical and 48 food isolates representing each of the identified PFGE patterns. Among each of the PFGE patterns, WGS analysis showed clinical isolates to be closely related genetically to food isolates. For most strains, there were less than four hqSNP differences between isolates; for strains serotyped as *Salmonella* Thompson, the difference was between 0 and 13 hqSNPs. All PFGE patterns involved in these outbreaks were either novel to the PulseNet database or identified previously only rarely (suggesting they were all very rare).

### Control measures

During the outbreak, three firms issued recalls for three brands of Maradol papayas sourced from Farm A in Mexico on 26 July, 5 August and 9 August. Products from Farms B and C were not recalled because the shipments associated with *Salmonella*-contaminated papayas were destroyed, and the previous shipments from these firms were past shelf-life by the time sample results were available. On 10 September 2017, one firm recalled Maradol papayas from Farm D in Mexico, since contaminated papayas were still on the market.

At the time the investigation began, all firms associated with papaya samples which yielded *Salmonella* were on Import Alert 21-17's ‘green list,’ meaning they had previously satisfied the requirements to be removed from the import alert. During the investigation, these firms were removed from the ‘green list’. Additionally, firms implicated in these outbreaks were added to the ‘red list’ for a different import alert, updated in 2017. This alert calls for the detention of fresh produce that appeared to have been prepared, packed or held under insanitary conditions and is updated as needed [22]. Firms placed on red-list status must submit sufficient evidence of corrective actions to FDA to be removed from this list [22].

## Discussion

Four concurrent multistate outbreaks of *Salmonella* infections were linked to imported Maradol papayas from four geographically dispersed Mexican farms in 2017. This was the first time papayas have been implicated as a multistate outbreak vehicle since the 2011 *Salmonella* Agona outbreak, and the first time four papaya-associated outbreaks were identified in 1 year, one of which was the largest papaya outbreak identified to date in the United States. The identification of multiple outbreaks and multiple implicated papaya farms highlights the potential for local contamination of an imported produce product like papayas to lead to widespread, disseminated illness in the United States. Collaborative work of local, state and federal public health and regulatory partners was instrumental in identifying the source of these outbreaks and preventing additional illnesses. These outbreak investigations demonstrate that industry vigilance and dedication to providing a safe product must be maintained long after issues have been identified and addressed, and that FDA import alerts and initial microbial testing alone are not always enough to prevent outbreaks from recurring. This is particularly relevant considering that there was not a sole supplier linked to all outbreaks.

Quick and targeted sampling of suspected foods during outbreak investigations, based on identifying common foods and sub-cluster locations, can provide crucial clues to identifying outbreak sources. Early in the *Salmonella* Kiambu investigation, interview data alone were not sufficient to identify a food item responsible for the outbreak, but a common retail sub-cluster was identified. By isolating the outbreak strain from targeted retail sampling of papayas, investigators were able to confirm papayas were the source of the illnesses. In 2017, sampling of papayas was essential for solving the originally identified cluster of *Salmonella* Kiambu infections and in identifying additional *Salmonella* strains and outbreaks. In the United States, we have a highly trained network of foodborne outbreak investigators around the country who are able to rapidly respond to foodborne outbreak investigations. Programmes such as FDA's Rapid Response Teams and CDC's OutbreakNet Enhanced programme are designed to support the ability of local and state health and regulatory agencies to rapidly respond to foodborne outbreaks [23, 24]. It is important to maintain and expand such programmes to strengthen our ability to respond to foodborne outbreaks around the country.

These outbreaks are examples of complicated investigations where multiple, cross-cutting investigation tools and strong collaboration are necessary to identify the source. Traditional epidemiological tools, such as the NHGQ, proved ineffective at identifying a source among multiple suspected food vehicles early in the investigation. During early hypothesis generation the percentage of case-patients reporting consumption of mangoes was slightly higher than the percentage reporting papaya consumption (50% *vs.* 42%), and both were higher than expected background rates for those foods, making it difficult to identify which food was most likely to be the source of the outbreak. Additionally, there are often higher overall consumption rates among case-patients for implicated produce items during multistate *Salmonella* outbreaks, in recent years ranging from 69% to 89% for outbreaks associated with cantaloupe, cucumbers and mangoes compared with 57% for the 2011 *Salmonella* Agona outbreak associated with papayas and 55% for the outbreak described in this paper, suggesting that papayas may be a food item that is more difficult to identify through epidemiological investigations than other produce items [10, 11, 13–15]. Smaller overall consumption rates for suspected food items and having multiple food items reported above expected background levels limit the ability of investigators to develop hypotheses based on early epidemiological data. Rather than relying solely on early epidemiological data to inform hypothesis generation, it is essential to evaluate epidemiological data along with laboratory and traceback data to determine the source of outbreaks.

Additionally, it would be beneficial to develop predictive epidemiological tools that could assist with identifying potential outbreak vehicles early in investigations. For example, a tool that could generate a list of potential vehicles based on early case-patient demographic information, such as race, ethnicity and gender, could help narrow the focus of an epidemiological investigation. One such pilot model has been developed using retrospective data for Shiga toxin-producing *Escherichia coli* outbreaks in the United States, which demonstrated the feasibility of using case demographic and outbreak characteristics to assist in predicting food sources [25]. However, more work is needed to develop and validate a tool that can be used with data that is readily available early in an investigation and adapt it for use with other pathogens, such as *Salmonella* [25]. Additionally, information on food import and distribution patterns within the United States coupled with geographical information for early outbreak case-patients could inform hypothesis generation. An analysis of historical import data for five produce-associated outbreaks demonstrated that the geographical distribution of illnesses differed between produce vehicles which were grown domestically compared with those imported into the United States from Mexico and Central America. Investigators concluded that the geospatial distribution of early illnesses might be useful in identifying suspected ports of entry for produce-associated outbreaks [26]. Targeted sampling of imported produce items from ports of entry, coupled with focused traceback investigations might accelerate the identification of outbreak vehicles during multistate outbreak investigations.

These four outbreaks highlight important steps investigators can take now and in the future to more rapidly and effectively respond to multistate foodborne outbreaks. Maintaining and expanding capacity building programmes around the country will assist investigators to more quickly solve future outbreaks. It may also be useful to increase targeted product testing during outbreak investigations to help find the source of illnesses, such as at points of services of interest identified through early epidemiological investigations and at certain ports of entry based on early case-patient locations. Novel hypothesis generation tools could also be developed to assist investigators in finding signals early in data collection, such as predictive models which generate potential food vehicles based on available early demographic and geographical data. The strengthening of current investigational tools along with the development of new tools could help in the evaluation of epidemiological, laboratory and traceback evidence together during investigations, to more quickly solve and stop outbreaks.
